# Supplementary material for: Are carbon emissions trading and green financial instruments synergistic? -Comprehensive quantitative research based on content analysis
Source: PLoS One. 2024 Mar 7;19(3):e0298601. doi: 10.1371/journal.pone.0298601 (PMC10919601; doi:10.1371/journal.pone.0298601)
Supplement: S1 Data — (ZIP) [file pone.0298601.s001.zip › Data/地级市Z_F工作报告.zip!/║╙▒▒/▓╫╓▌╩╨/└┤╫╘╣1⁄2╓┌║┼í╛┬φ┐╦╩2╛▌═°í┐_2015.docx]

各位代表：

现在，我代表沧州市人民政府向大会作工作报告，请予审议，并请各位政协委员和列席会议的同志提出意见。

一、2014年工作回顾

过去一年，在中共沧州市委的坚强领导下，在市人大、市政协及社会各界的监督支持下，我们紧紧围绕打造河北沿海地区率先发展增长极目标，统筹推进“三大经济板块”，着力稳增长、调结构、促改革、惠民生，较好地完成了十三届人大二次会议确定的各项任务目标。全市完成地区生产总值3133.38亿元，增长8%；固定资产投资2728.9亿元，增长19.1%；公共财政预算收入189.7亿元，增长10.1%；规模以上工业增加值1302.8亿元，增长9.2%；城镇居民人均可支配收入25059元，农民人均纯收入9592元，分别增长9.5%和11.5%。主要指标增速保持全省前列，经济运行质量进一步提高。

（一）沿海开发建设取得新突破。黄骅港20万吨级航道、20万吨级矿石码头和邯黄铁路建成运营，如期实现“三同步”。河北钢铁通用散货、美国嘉吉粮油、浙江泰地液体化工等7个在建码头顺利推进，煤炭港区四期工程通过验收。全年完成港口吞吐量1.75亿吨，集装箱31.4万标箱，集装箱吞吐量增长36.29%。临港产业加速聚集，中海油中捷石化炼化一体化、华泰万国石材城等在建项目稳步推进，河北中重高端冷轧板、神华机车检修和正元化肥60万吨合成氨、80万吨尿素等项目竣工投产。全年新增物流贸易企业530家，贸易额和税收增幅均超过50%，港口物流成为沿海经济发展的新亮点。

（二）中心城区经济充满活力。以“三区两园”为平台，大力发展新型工业，森茂特激光、东塑新能源锂电池隔膜、意大利蒙特费罗导轨机械等一批高新技术和高端装备制造项目竣工投产。着力推进商贸服务业拓展升级，明珠商贸城、义乌小商品城、荣盛购物广场建成运营。华信现代农业物流配送中心、沃尔玛购物中心扎实推进。北京路18个企业总部主体封顶，张家口银行、华夏人寿保险等5家银行和保险公司落户沧州。中心城区公共财政预算收入占全市比重超过1/3，沧州连续两年跻身福布斯中文版中国大陆最佳商业城市百强。

（三）县域经济不断发展壮大。支持县域依托优势产业加快转型升级步伐，促进县域工业增投入、提效益、上水平。全市销售收入超百亿元产业集群达到12个，龙头示范企业43家，居全省第一。盐山、河间评为“河北省特色产业名县”，泊头获“中国大气污染治理装备制造产业基地”称号，任丘位列中国中小城市综合实力百强县（市）前50强。吴桥中汽轮胎、肃宁华斯产业园二期、河间奥丁新能源中央空调等142个亿元以上项目开工建设，东光化工、献县日新易拉罐等153个项目竣工投产。全市新增上市企业18家，总数达到70家，其中，县域新增15家，总数57家，在华北地区率先实现上市公司县域全覆盖。

（四）重点项目建设快速推进。紧紧抓住大项目、好项目不放手，深入实施重点项目领导分包责任制和“4+2”观摩考评机制，一批重大战略支撑项目取得突破性进展，为未来发展奠定了坚实基础。总投资101亿元的华北石化千万吨炼油项目全面开工；全省单体规模最大、总投资1200亿元的海兴核电和一期投资400亿元的核燃料产业园项目，签订战略合作协议；年产30万辆整车、总投资120亿元、世界一流的北京现代四工厂项目于2014年12月29日签约，今年3月28日开工建设。全年新开工亿元以上项目216个，竣工投产212个；新增“四上”企业516家，居全省第一。项目建设势头足、成效好，全省重点项目观摩调度会在我市召开，更加坚定了我们加快项目建设的信心和决心。

（五）工业经济实现提质增效。努力克服经济下行压力大、企业经营困难等不利因素，加大对重点企业的指导和生产要素的协调调度，工业经济实现平稳回升、较快增长。规上工业增加值增速全省第一，总量跃居全省第三，规上工业企业总数突破2000家。扎实推进技术改造和百强培育工程，190个亿元以上技改项目累计完成投资332.9亿元，百强企业完成主营业务收入和税金占全市规上工业的比重达到46.8%和76%。新增高新技术企业23家、科技型中小企业1680家，总数分别达到111家和2500家，高新技术企业完成增加值230亿元，增长15%。工业园区建设成效突出，省级以上开发区主营业务收入、工业增加值、固定资产投资等主要指标增幅均超过20%。主营业务收入超百亿元开发区达到17个，其中，临港经济技术开发区超过500亿元，任丘开发区突破1000亿元。

（六）城镇化建设迈出新步伐。围绕完善城市功能、提升城市承载力，实施了一批重点城建项目。沧州体育场、游泳馆、博物馆建成投用，黄河路城市快车道、吉林大道等12条城市道路竣工通车，41条小街小巷完成改造任务。水文四队、后辛庄、大杨庄等10个棚户区改造项目稳步实施，皇家壹里、锦绣天地等住宅小区交付入住。完成16个老旧小区二次管网改造，东部城区新增集中供热面积270万平方米，运东垃圾发电厂正式运行。深入开展环境提升年活动，店外经营、露天烧烤、环境卫生、交通秩序得到有效整治和改善。大力实施绿色行动计划，千童、永济等10个街头游园，长芦大道、黄河西路等10条绿化景观带如期完工，新增绿化面积866公顷，顺利通过国家园林城市初评。县城建设全面提速，综合承载能力大幅提升，肃宁通过国家级园林县城初检，任丘、献县成功创建省级园林县城，吴桥、献县荣获省“人居环境进步奖”称号。全市城镇化率预计达到46.5%。

（七）农业农村稳定发展。粮食生产再获丰收，总产达到89.8亿斤。渤海粮仓科技示范工程稳步实施，推广示范面积60万亩，增产1亿斤。积极推进农业产业化经营，献县千隆150万头生猪屠宰加工、中捷犇放牧业5万头奶牛养殖等一批大型现代农业项目建成投产，新增省级龙头企业15家，总数达到59家，产业化经营率达到58.7%。新增农民专业合作组织3636家，总数达到8631家。16个试点村全部完成农村土地承包经营权确权登记颁证工作。全市新增土地流转面积21万亩，总数达到159万亩。进一步加大农业基础设施投入，发展节水灌溉面积70万亩，新修农村公路638.7公里，新建和改造10千伏农网线路1677公里，配变站2463台。美丽乡村建设成效显著，243个省级重点村全部完成改造提升任务。

（八）改革开放稳步推进。持续深化改革、扩大开放，发展动力和活力进一步增强。深化商事登记制度改革，新增各类市场主体4.7万户，增长26.6%，总数达到22.7万户。行政审批制度改革深入推进，衔接取消下放行政审批事项162项，市级行政审批事项由449项精简到274项，精简率达39%。市政公司等市属国有集体企业改革顺利完成，渤海新区成功回购沧盐集团股权。对接京津取得明显成效，共实施京津项目775个，总投资1146亿元。北京现代四工厂、北京生物医药产业转移基地等项目签订入区协议。由沧州始发至北京的高铁列车开通运行。“中国人民解放军总医院（北京301医院）沧州合作医院”顺利揭牌。中捷中欧产业园正式成立，并被省政府确定为河北面向中东欧开放合作的重要平台。成功举办韩国首尔、北京中关村投资环境说明会等主题招商活动。全年直接利用外资3.4亿美元，引进省外资金710亿元，完成外贸进出口31亿美元。

（九）民生和社会事业全面进步。“十大民生工程”扎实推进，顺利完成既定任务目标。保障性住房分配入住2.2万套，改造农村危房7759套，近3万户城乡居民乔迁新居。全市城镇新增就业6.18万人，农村劳动力转移就业5.4万人，城镇登记失业率控制在3.5%以内，高校毕业生就业率达到96%。适度提高了机关事业单位津补贴标准。企业退休人员养老金月增182元，城乡居民低保和医疗补助标准分别达到5400元、2500元和320元，城镇基本医疗保险、新农合、城乡居民养老保险参保率达到96%以上。投入扶贫开发资金33亿元，8.4万人稳定脱贫，吴桥、东光、孟村提前脱贫出列。沧州一中新校区竣工投用。“暖心工程”让83万学生喝上放心热水。成功承办河北省第十四届运动会，参赛成绩实现历史性突破，位列实得金牌榜第四位。启动实施“单独两孩”政策。乡村卫生服务一体化管理全面推开，全市31所县级公立医院全部纳入综合改革范围，其中8所列入国家改革试点单位。南水北调工程保沧干渠全线贯通，石津干渠完成总任务量的75%，水厂以上输水管道工程全部开工。地下水超采综合治理试点工作全面启动。63.5万人饮水安全问题得到解决。深入实施大气污染防治行动计划，淘汰改造燃煤锅炉325台，压减燃煤33.4万吨，淘汰黄标车5.6万辆，更新燃气公交车1165部，PM2.5浓度下降13.7%，主要污染物减排任务全部完成。沧州市区空气质量达标天数增加11天，总数达到144天。全市新增植树造林面积35.1万亩，森林覆盖率达到25.5%。“双拥”共建工作不断加强，圆满完成退役士兵安置和征兵工作，连续四届获得“省级双拥模范城”称号。扎实推进平安沧州建设，食品药品安全监管力度不断加大，安全生产、社会治安形势总体稳定。

（十）政府自身建设持续加强。深入开展党的群众路线教育实践活动，大力改进工作作风，政府工作人员的宗旨意识、服务能力不断提高。加快政府职能转变，推进“三级平台”建设，完善“两个代办”机制，积极构建便捷高效的为民服务网络，服务型政府建设迈出新步伐。坚持科学民主决策，深入推进依法行政，主动向人大报告工作、向政协通报情况，全年共办理人大代表建议202件、政协提案413件，按时办复率100%。严格落实党风廉政建设责任制，深入开展正风肃纪专项行动，强化经济责任审计，加大案件查处力度，政府廉政建设取得新成效。

各位代表，过去一年，面对各种困难和挑战，我们牢牢把握发展第一要务，壮大经济总量，提升发展质量，成功实现“弯道超车”，地区生产总值跃居全省第三位，全部财政收入保持全省第三，经济实力跻身全省第一梯队；我们坚持把项目建设作为推动发展的第一抓手，以项目论英雄，以项目促发展，一批既利当前、更惠长远的重大战略支撑项目取得历史性突破；我们始终把730万沧州人民的幸福期待作为政府工作的努力方向，着眼事关群众切身利益的热点难点问题，持之以恒抓落实、求实效，汇聚了谋事干事、善作善成的正能量，巩固发展了社会和谐稳定的大好局面。

各位代表，成绩来之不易，奋斗历尽艰辛。这些成绩的取得，是市委正确领导的结果，是市人大、市政协鼎力支持的结果，是全市人民团结一心、攻坚克难的结果。在此，我代表沧州市人民政府，向辛勤工作在各条战线上的全市各族人民，向给予我们大力支持的人大代表、政协委员和各民主党派、工商联、无党派人士，向离退休老干部、驻沧各单位、人民解放军和武警官兵，向所有关心、支持沧州发展的同志们、朋友们，致以崇高的敬意和衷心的感谢！

在总结成绩的同时，我们也清醒地看到经济社会发展中的困难和问题。主要是：传统产业转型升级任务重，创新能力不强，高新技术产业和现代服务业尚未形成有效支撑；城乡发展不均衡，城镇化率低于全省平均水平；资源环境容量约束加剧，节能减排和生态环境保护任务艰巨；经济外向度和利用外资规模与沿海城市区位不匹配；个别部门和工作人员服务意识差、工作效率低，依法行政能力有待进一步提高。对于这些问题，我们要采取有效措施，认真加以解决。

二、2015年总体要求

今年是全面完成“十二五”规划的收官之年，是全面深化改革的关键之年，是全面推进法治沧州建设的开局之年，做好2015年经济社会发展的各项工作，意义十分重大。

纵观当前国内外发展形势，挑战与机遇并存。世界经济缓慢复苏，但总体增长疲弱态势难有明显改观；我国经济处于“三期叠加”的特殊阶段，经济下行压力依然较大。但从长远看，我国进入经济发展新常态，速度更趋平稳、动力更为多元、结构更加优化，具有巨大的韧性、潜力和回旋余地，仍处于可以大有作为的重要战略机遇期。沧州区位独特，优势明显，蕴含着诸多有利发展的积极因素。特别是随着京津冀协同发展与《河北沿海地区发展规划》两大国家战略的深入推进，一大批战略支撑项目的跟进实施，我市的发展潜力将进一步释放，后发优势将进一步彰显，具备了“稳”的坚实基础和“进”的有利条件。我们既要增强忧患意识，把困难和挑战估计得充分一些，更要坚定发展信心，抢抓发展机遇，唱响“大开放、大开发、大建设、大发展”主旋律，奋力开创经济社会发展新局面！

政府工作的总体要求是：全面贯彻党的十八大和十八届三中、四中全会，中央和省、市经济工作会议精神，紧紧围绕打造河北沿海地区率先发展增长极和京津冀区域新的增长极目标，坚持稳中求进工作总基调，主动适应经济发展新常态，以提高发展质量效益为中心，以改革开放为动力，大力实施创新驱动战略，稳增长、调结构、上项目、惠民生，促进经济社会平稳较快发展，加快建设创新之城、产业之城、文化之城、生态之城、好人之城。

全年经济社会发展的主要预期目标是：地区生产总值增长8%，全部财政收入增长7%，公共财政预算收入增长8%，固定资产投资增长20%，规模以上工业增加值增长9%，服务业增加值增长9%以上，直接利用外资增长10%，城乡居民人均可支配收入增长9%，社会消费品零售总额增长12%，人口自然增长率控制在8.7‰以内，单位生产总值能耗和主要污染物排放达到省控制目标。今年主要指标的设定，增幅均高于全省平均水平，特别是固定资产投资增长20%，既充分考虑了今年的宏观经济形势，又兼顾了我市的发展基础和条件；既贯彻了稳中求进的工作总基调，又体现了打造增长极的现实需要，有利于调动全市上下的积极性和能动性，打造沧州科学发展、绿色崛起的“升级版”。

在实际工作中，我们将注重把握好以下六个方面：一是更加注重经济运行的质量和效益，努力实现新常态下又好又快发展。顺应速度变化、结构优化、动力转换的趋势性变化，更加积极主动地调结构、转方式，改造提升传统优势产业，培育发展战略性新兴产业和现代服务业，推动发展质量、效益不断提升，速度、规模较快增长。二是更加注重创新驱动，增强经济发展的内生动力。搭建创新平台，完善创新政策，丰富创新手段，破解创新瓶颈，推进全民、全域、全方位创新。突出抓好科技、人才、金融、管理等关键领域改革创新，提升科技创新对经济增长的贡献率。三是更加注重利用社会资本，激发市场主体活力。着力消除体制机制障碍，打破束缚民资进入的“天花板”，构建服务市场主体的“助推器”，引导支持各类社会资本投向实体、活跃经济。四是更加注重对接京津，全面融入协同发展大格局。找准对接着力点、切入点和共赢点，促进规划衔接、产业对接、功能承接、交通联接，推动与京津合作取得更大突破。五是更加注重生态环境建设，加快打造生态沧州、美丽沧州。强化生态意识、环保意识，以更加有力的举措，扎实推进大气污染防治、水生态保护和绿化造林工作，让全市人民享受碧水蓝天，呼吸到新鲜空气。六是更加注重民生保障，解决好群众诉求和关切。认真办好群众衣食住行以及就业、教育、文化、医疗、养老等惠民利民实事，努力为全市人民营造工作安心、生活舒心、办事顺心、安定和谐的社会大环境。

各位代表，抢抓历史机遇，推进率先发展，既是省委、省政府的希望和重托，更是全市人民的厚望和期盼，只要我们坚定信心，全力攻坚，就一定能够圆满完成全年的任务目标！

三、2015年主要任务

重点抓好八个方面的工作。

（一）统筹推进“三大经济板块”，快速提升综合实力。坚持分类指导，统筹兼顾，协调推进沿海经济、中心城区经济和县域经济，形成多极支撑、多元发展的新格局。

全力推进沿海经济率先发展。深入实施沿海开放带动战略，推动工作重心从大规模基础设施建设向加速临港产业聚集转变，确保渤海新区固定资产投资增长30%。加快河北海伟液体化工、浙江和润粮油等系列码头建设，进一步提高港口运营能力，确保黄骅港口岸正式开放，力争综合保税区申报成功，全年完成吞吐量2.2亿吨，集装箱50万标箱。做大做强“九大功能园区”，突出抓好北京生物医药产业转移基地、航天科技煤制合成气等一批产业项目。大力发展物流贸易，培育优势物流企业，力争完成物流贸易额1600亿元。加快黄骅新城、南大港湿地国际旅游城、中捷欧洲特色街区建设，进一步完善道路管网、商贸餐饮、教育医疗、休闲娱乐等配套功能。积极推进石港、邯港高速公路尽快开工建设，加快石（衡）港城际铁路、首都第二机场至黄骅港城际铁路前期工作，抓好沧州到港口地方铁路客运开通工作，构筑对接京津、联通腹地、便捷畅通的综合交通体系。

促进中心城区经济突破提升。依托沧州开发区、沧东开发区、沧州高新区和新华、运河两区，大力发展高端制造业和现代服务业，进一步做强中心城区产业支撑。以北京现代四工厂、汽车配件产业园和核燃料产业园落户中心城区为重要契机，加快规划调整、征地拆迁、手续跑办、配套设施建设等相关工作，确保按时间节点强力推进。发挥沧州高铁站周边40平方公里区域功能完备、设施齐全、高科技含量高附加值项目可直接落地的优势，积极承接京津新能源、新材料、总部经济、文化创意、教育医疗等产业转移和科技成果转化，努力打造沧州现代产业基地，带动中心城区经济提速发展。

着力提高县域经济发展水平。强化县域工业主体地位，以园区建设为载体，发展壮大特色产业，推进项目向园区集中，园区与城镇融合发展，力争主营业务收入超200亿元园区达到8个，超500亿元3个。支持任丘加强与华北油田、华夏幸福基业战略合作，推进油地共建，打造白洋淀产业新城；支持盐山、孟村加快管道装备产业转型升级，做大规模，提升水平；支持肃宁、南皮、河间依托优势产业，推进裘皮城、五金城、冀中澳林博览城建设；支持青县、泊头、东光、献县进一步做强主导产业，提升品牌产品影响力；支持海兴大力发展绿色能源产业；支持吴桥加快打造旅游名县。

（二）突出创新驱动，打造经济增长新引擎。坚持把创新驱动贯穿于经济发展全过程，推动以科技创新为重点的全面创新，聚集创新资源，激发创新活力，着力打造“创新之城”。

培育激活企业创新主体。引导企业和社会资本加大研发投入，建立研发机构，完善研发体系。鼓励优势企业建立产业技术创新联盟，在精细化工、机械制造、智能装备、清洁能源、信息技术等领域，开展关键核心技术和重大科技成果联合攻关。对重大技术改造及研发项目给予优先支持，培育壮大一批具有创新引领能力和国际竞争力的旗舰型企业。鼓励企业实施产品创新、管理创新、技术创新，以创新提水平，以创新求发展。

建立健全科技创新机制。推进落实《鼓励和支持人才创业的十条措施》，积极推行科技成果产权制度改革、股权和分红激励等政策，促进技术资本化、人才股份化，全面激发各类人才创新创造活力。强化知识产权保护和应用，推动知识产权密集型产业发展。加快引进一批引领高新技术产业发展的高层次人才，吸引“两院”院士、京津研发机构带项目、带技术、带团队到我市创新创业。用足用好推向全国的6项中关村政策，打造自主创新示范区、创新驱动先行区。

实施“科技创新三年计划”。坚持培育、引进并重，力争用三年时间，实现“四个一百”目标，即：新增高新技术企业100家，全市高新技术企业数量和规模以上高新技术产业增加值实现翻番增长；搭建科技型中小企业成长阶梯，新增“科技小巨人”企业100家；推进产学研协同创新，新增市级以上企业孵化器、产业研究院、院士工作站、工程技术中心等公共技术创新服务平台100个；加快科技成果转化，实施国家级、省级科技创新项目100个。通过三年计划的推进实施，不断增强科技创新能力，为我市转型升级、绿色崛起提供有力支撑。

（三）做强项目支撑，夯实经济发展根基。坚持把投资和项目作为经济增长的第一拉动力，持续加大重点项目建设力度，为打造“产业之城”积聚新能量。

狠抓龙头项目带动。着眼发挥重大项目的裂变效应，全力抓好30个重大战略支撑项目，带动一批配套项目和产业集群迅速膨胀壮大。以华北石化千万吨炼油为龙头，带动后续80万吨PX、120万吨PTA等产业链项目，打造世界级石化产业基地；以海兴核电、核燃料产业园为龙头，加快前期工作，力争今年取得核准，早日开工建设，打造河北新型清洁能源基地；以北京现代四工厂30万辆整车为龙头，带动近百家汽车零部件企业和相关产业聚集发展，打造整车制造、出口及零部件加工基地；以华泰万国石材城为龙头，吸引更多石材加工销售企业集群发展，打造国内一流的石材集散基地；以中铁装备制造为龙头，推进实施300万吨冷轧薄板、300万吨超细粉等延伸配套项目，打造装备新材料生产基地。通过“五大龙头”的推进实施和后续项目的配套跟进，带动3000亿元的固定资产投资，形成5个主营业务收入超千亿元的产业园区。

狠抓项目谋划储备。按照“干净、高端”要求，深入谋划一批与京津合作项目、高科技含量项目、绿色循环低碳项目和后工业化项目，确保市级重点项目库储备超亿元项目1000个以上，超10亿元产业项目300个，30亿元产业项目30个，列入省重点建设项目30个，力争一批项目进入省“十三五”建设计划。

狠抓项目督导落实。深入实施重点项目领导分包责任制和“4+2”观摩考评机制，引导优质资源向重点项目、重点产业集中，土地、资金等要素向能开工、能竣工的项目倾斜。高度重视项目质量，着力推进一批产品档次高、产业链条长、附加值高的优质项目。年内新开工亿元以上重点项目200个，竣工100个，新增入统企业50家，力争产业项目投资增长率达到20%以上。

（四）调结构转方式，全面提升产业层次和水平。坚持把调结构、转方式放在更加重要的位置，通过优化存量，提升增量，推动经济提质增效、转型升级。

改造提升传统产业。坚持“有中生新”，推动传统产业向中高端迈进，向产业链上下游延伸，“让老树开出新花”。重点抓好华北石化、中捷石化、鑫海石化、沧炼公司等骨干企业炼油质量升级改造，大幅提升产品竞争力，向绿色、低碳、循环经济方向发展。推进装备制造业顺应智能化、高端化趋势，由零部件加工向成套整机转型，由规模扩张向技术创新、优质高效转型。重点抓好河北三和重工、达力普石油专用管等150个亿元以上技改项目，推动企业提档升级，占领高端市场。

着力培育壮大新兴产业。坚持“无中生有”，推进电子信息、新能源、新材料、节能环保、生物制药等战略性新兴产业规模化、聚集化发展，“让新芽长成大树”。依托渤海新区海洋经济产业园、北京生物医药产业园、化工新材料产业基地和沧州高新区、中捷高新区，推动实施华晨海洋生物医药、中捷通航专用设备、中国机械机器人、航天神州太阳能等重点项目，催生一批优势产品，形成一批特色产业集群。

大力发展现代服务业。像抓工业一样抓服务业，集中力量培育一批有特色、有规模、有影响力的现代服务业主导产业。坚持生产性服务业和生活性服务业并重，大力发展现代物流、金融保险、信息服务、电子商务、文化旅游、健康养老等现代服务业，促进云计算、大数据、物联网与制造业融合发展。以渤海新区、中心城区和肃宁三大物流聚集区为平台，以“外引”促“内生”，做大做强现代物流业。鼓励支持域外银行、证券、保险公司在沧设立分支机构，进一步丰富金融业态。加快发展总部经济，培育一批税收超千万元的商务楼、写字楼。依托运河、湿地、武术、杂技等文化旅游资源，积极引进华谊兄弟、深圳华强等有实力的文化企业集团，建设一批特色鲜明的文化产业园和文化创意产业基地，推进沧州“文化之城”建设。

积极推进质量强市建设。强化质量兴市、质量强市理念，组织实施“全国质量强市示范城市”创建活动，全面提升产品、工程、服务、环境四大质量水平。发挥产品质量安全导向作用，大力开展产品质量提升行动和质量检验检测中心建设，规范、提升特色优势产业，推进企业产品向标准化、品牌化方向发展。

做强骨干龙头企业。加强企业家培训与队伍建设，培养和造就一批具有战略眼光和现代经营管理理念的高素质企业带头人。对销售收入多、税收贡献大、社会信誉好的工业、服务业“双百强”企业，在项目用地、贷款融资、要素保障、市场开拓等方面加大帮扶力度，促其做大规模、做强实力，打造一批主营业务收入超50亿元、过100亿元的行业龙头。积极推动优势骨干企业上市融资，力争每个县（市）新增一家上市企业。

（五）推进新型城镇化建设，促进城乡统筹协调发展。坚持以人的城镇化为核心，进一步做大城市规模、做优城市功能、做美城市环境，打造靓丽优美、宜居宜业的“生态之城”。

着力提升中心城区承载能力。围绕创建国家园林城市，着眼东西部城区均衡发展，进一步完善城市功能、提升城市品位，推进实施一批重点城建工程。创建国家园林城市工程：以“绿”为根，以“水”为魂，突出做好水、绿两篇文章。改造新建人民、双金等7个公园和3个游园，抓好8条道路绿化，中心城区新增绿化面积500万平方米。积极推进御河景观带一期工程，带动整体片区开发，把大运河沿岸打造成为高品质的城市景观和文化旅游走廊。加快环城林带建设和小流津河等12条排干河渠疏通改造，构筑绿色屏障、生态水网。城市基础设施工程：做好主城区地下供排水、燃气、热力、电力、通信等8大类20余种管线普查工作。重点抓好37个老旧小区二次管网改造、西部城区调峰锅炉2个集中供热项目、运西污水处理厂二期建设。道路互联互通工程：谋划实施307国道改线、永济路跨京沪铁路桥等重点项目，新建黄河路东延等4条城市道路，改造小街小巷19条，新建26个公交港湾，优化调整45个公交站点，建设便捷畅通的城市交通网络。棚户区改造工程：用足用好国家相关优惠政策，重点抓好流津时代、东方世纪家园等7个国开行贷款项目，确保芦园村等3个棚户区改造和商业住宅项目顺利推进，进一步改善市民居住条件。

大力推动县城建设上水平。合理优化县城空间布局，依托老城，开发新城，集中优质资源，建设高水平的公共服务和大型商贸设施，打造一批标志性建筑和特色空间节点，推动县城向各具特色、功能完善的中小城市发展。完善县城规划体系，加快“多规合一”步伐，将县城周边3—5公里区域纳入规划管理范围，促进县城扩容提质，增强对产业和人口的吸纳、承载能力。抓好县城周边小城镇的统筹规划和配套功能建设，建成一批服务县城、带动农村的产业重镇、商贸强镇、旅游名镇。

切实加强城市综合管理。巩固中心城区环境提升年治理成果，拓展数字化城管服务功能，推进城市管理标准化、精细化、长效化。加强智慧城市建设，促进信息化与城市发展深度融合，打造全省智慧城市典型示范。开展城镇环境容貌整治活动，加大治违、治脏、治乱和建设投入力度，提高县城、乡镇规范化管理水平。

（六）优先发展现代农业，着力做好“三农”工作。坚持农业基础地位不动摇，加快转变农业发展方式，进一步提高农业产出效益，持续增加农民收入。

着力推进农业产业化。以提高农业综合生产能力、投入产出效益为核心，推进农业产业化、现代化。大力培育和引进一批品牌龙头企业，重点抓好“10大集团”和“50强”企业晋档升级。积极扶持农民专业合作社、家庭农场、专业大户发展规模经营和精深加工，打造覆盖面广、带动力强、效益明显的产业链条。力争产业化经营率提高1.5个百分点，达到60%以上。

着力发展绿色、高效农业。深入实施渤海粮仓科技示范工程，完成示范推广面积100万亩，增粮2亿斤。抓住地下水超采综合治理的有利时机，加快实施一批农业节水灌溉项目。依托畜牧、果品、蔬菜等优势产业，加强标准化规模种养殖示范区建设，瞄准京津市场，做响青县蔬菜、泊头鸭梨、沧县红枣等农业品牌，打造京津农副产品生产供应基地。

着力改善农村生产生活条件。以道路硬化、垃圾处理、村庄绿化等6项工作为重点，深入开展农村面貌改造提升行动，完成300个省重点村的改造提升任务。实施农村公路危桥三年改造计划，保障农村公路安全。充分利用省定扶贫政策，全面落实扶贫开发计划，重点推进“牧菜菌”三大扶贫产业，打造2—3个蔬菜、食用菌基地县，稳定解决8万农村贫困人口的增收脱贫问题，确保献县脱贫出列。鼓励农村剩余劳动力向非农领域转移，不断增加农村人口工资性收入。

（七）深化改革开放，为发展增动力添活力。坚持深入推进重点领域、关键环节改革，进一步扩大对内对外开放，以改革的新突破、开放的新成效推动经济社会持续健康发展。

加快转变政府职能。大力推进简政放权，衔接落实国务院和省政府下放行政审批事项，清理非行政许可审批事项，研究制定权力清单、责任清单和负面清单，推广网上并联审批新模式。抓好渤海新区行政审批局试点工作，推进向城区下放部分经济管理权限、向三个开发区下放行政审批事项。积极稳妥推进政府机构改革，调整理顺行政管理体制。率先在全省实施工商营业执照、组织机构代码证和税务登记证“三证合一”登记制度改革。进一步发挥市政务服务中心、公共资源交易中心和金融服务中心作用，提升各县（市）政务服务中心服务能力，完善“三级平台”、“两个代办”机制，不断提高行政效能和服务水平。

推进重点领域改革。加快国有集体企业公司制改革和企业集团化步伐，重点抓好市环资公司、车辆厂等10家企业改革工作。进一步激发民营经济活力，落实扶持政策，改善创业环境，营造大众创业、万众创新的浓厚氛围，力争新增市场主体5万个。深化农村综合改革，稳步推进土地承包经营权确权登记试点工作，引导规范农村土地经营权依法有序流转。抓好青县、泊头、肃宁三个省级农业农村改革试点，为全市农村改革探索经验。深化财税体制改革，推行全面规范、公开透明的预算管理制度，推广政府与社会资本合作（PPP）模式，发展股权和创业投资基金，最大限度地发挥财政资金效益。加强地方政府债务管理，强化投资公司、金融中介机构监管，防范和化解债务金融风险。统筹推进教育、医疗、文化等领域改革。

扩大对内对外开放。充分发挥自身优势，推动更高层次“引进来”，更大步伐“走出去”，打好利用外资翻身仗。突出园区招商主体作用，创新招商方式，实施精准招商，提高招商引资的针对性和成功率。抢抓京津冀协同发展重大机遇，瞄准京津大型央企、国内外500强、行业龙头及各类商会、协会，加强高层对接，洽谈引进一批大项目、好项目。以日韩、德国和中东欧国家为重点，精心谋划组织经贸洽谈活动，深度推介包装项目，努力在引进外资上取得更大突破。加快中捷中欧产业园建设，打造全省对外开放的新亮点、中东欧国家合作的示范区。稳步扩大外贸出口，加强综合保税区和外贸转型示范基地建设，培育出口竞争新优势。进一步扩大服务外包、文化贸易、技术进出口规模，不断拓展外经发展空间。

（八）加强民生保障，全面发展社会各项事业。坚持把保障和改善民生放在心上、抓在手上，持续加大民生投入，不断提高人民群众的幸福感和满意度。

健全完善社会保障体系。推进完善“五险合一”管理模式，开展机关事业单位养老保险制度改革，继续提高企业退休人员和城乡居民养老金、工伤保险待遇标准。加大城乡养老助残力度，实施贫困残疾儿童免费抢救性康复工程。加强保障性安居工程建设。建设棚户区改造和保障性住房2.04万套。努力解决就业问题。实施更加积极的就业扶持政策，统筹解决好高校毕业生、城镇困难人员、退役军人等重点人群就业问题，确保全市新增就业5.5万人，城镇登记失业率控制在4.5%以内。优化教育资源配置。加强学前教育和中小学教师队伍建设，提高教育教学质量，创建市级一类以上幼儿园55所、标准化中小学50所。大力发展现代职业教育，推动高校内涵式发展。加快市十三中、沧州工贸学校扩建搬迁工作。繁荣文化体育事业。积极做好第十五届中国吴桥国际杂技节申报承办工作。加强公共文体设施建设，满足群众多层次、多样化的精神文化和健身娱乐需求。提升医疗卫生服务水平。加强与国内大型医疗机构合作，加大社会办医力度。健全乡村、社区卫生服务体系，鼓励市、县公立医院向基层延伸服务。支持发展中医药事业，提升中医药服务能力。做好引蓄水和农村饮水安全工程。加快南水北调配套工程建设，完成水厂以上输水管道工程。用足用好引黄指标，力促李家岸、小开河引黄线路开工。加强坑塘、沟渠水系治理，提高引蓄水能力。继续实施农村饮水安全工程，解决50万人饮水安全问题。持续改善生态环境。扎实开展地下水超采综合治理工作，压采深层水1.47亿立方。深入实施新《环境保护法》，打好大气污染防治攻坚战，确保完成省政府责任书各项任务目标。推进造林绿化工程，完成造林面积40万亩，森林覆盖率达到27.5%。深入推进平安沧州建设。贯彻落实新《安全生产法》，严格落实安全生产“党政同责、一岗双责”，构建齐抓共管的安全监管大格局。深入开展专项整治行动，确保人民群众饮食和用药安全。进一步创新社会管理，提高快速反应和社会管控能力，依法严厉打击各类违法犯罪活动，切实维护社会平安稳定。大力弘扬社会主义核心价值观，不断加强公民道德建设，叫响沧州“好人之城”品牌。

加强国防教育和后备力量建设，巩固增进军政军民团结。支持工会、共青团、妇联等人民团体发挥桥梁纽带作用，做好民族宗教、外事侨务、妇女儿童、防震减灾、史志档案、红十字会等工作。高质量编制“十三五”规划，使之成为体现国家战略、符合沧州实际的好规划。

四、加强政府自身建设

面对新的形势和任务，我们将按照为民、务实、清廉的要求，进一步加强政府自身建设，全面提升政府工作水平。

提高依法行政能力，建设法治政府。深入落实《全面推进法治沧州建设的实施意见》，严格按照法定权限行使权力、履行职责。自觉接受人大法律监督、政协民主监督。进一步健全决策机制，完善决策程序，不断提高政府决策的科学化、民主化水平。深入推进政务公开、信息公开，让人民赋予的权力在阳光下公开透明运行。

提高服务群众能力，建设高效政府。大力改进工作作风，深入基层，听取民意，多谋民生之利，多办惠民之事，密切同人民群众的血肉联系。加强行政效能监察，优化政务服务，建立管理规范、运行高效的政务服务体系，以良好的政风和实实在在的行动赢得企业的信赖和人民的支持。

提高干事创业能力，建设务实政府。加强教育培训，实施素质提升工程，提高政府公务人员新常态下推动工作、促进发展的能力和水平。开展深度对标，学习借鉴先进地区的开放理念和创新办法，推动重大决策实施、重点领域突破。加强执行力建设，对确定的目标任务，敢打攻坚战，敢啃硬骨头，一抓到底，久久为功，确保各项工作不折不扣落到实处。

提高廉洁从政能力，建设清廉政府。严格落实中央“八项规定”，自觉践行“三严三实”要求，持续整治“四风”，巩固和扩大党的群众路线教育实践活动成果。严格落实党风廉政建设责任制，深入推进政府系统预防和惩治腐败体系建设，打造一支既干净、又干事，既谋事、又成事的公务员队伍，让为民、务实、清廉成为广大党员干部的自觉行动。

各位代表！建设沿海强市、美丽沧州的光荣使命鼓舞我们砥砺奋进，全市人民的信任期盼鞭策我们勇往直前。让我们在中共沧州市委的正确领导下，戮力同心、埋头苦干，为实现沧州科学发展、绿色崛起而努力奋斗！
